# Deterministic theory of evolutionary games on temporal networks

**DOI:** 10.1098/rsif.2024.0055

**Published:** 2024-05-29

**Authors:** Xiaofeng Wang, Feng Fu, Long Wang

**Affiliations:** ^1^ Department of Automation, School of Information Science and Technology, Donghua University, Shanghai 201620, People's Republic of China; ^2^ Engineering Research Center of Digitized Textile and Apparel Technology (Ministry of Education), Donghua University, Shanghai 201620, People's Republic of China; ^3^ Department of Mathematics, Dartmouth College, Hanover, NH 03755, USA; ^4^ Department of Biomedical Data Science, Geisel School of Medicine at Dartmouth, Lebanon, NH 03756, USA; ^5^ Center for Systems and Control, College of Engineering, Peking University, Beijing 100871, People's Republic of China

**Keywords:** evolutionary game theory, temporal networks, deterministic dynamics

## Abstract

Recent empirical studies have revealed that social interactions among agents in realistic networks merely exist intermittently and occur in a particular sequential order. However, it remains unexplored how to theoretically describe evolutionary dynamics of multiple strategies on temporal networks. Herein, we develop a deterministic theory for studying evolutionary dynamics of any n×n pairwise games in structured populations where individuals are connected and organized by temporally activated edges. In the limit of weak selection, we derive replicator-like equations with a transformed payoff matrix characterizing how the mean frequency of each strategy varies over time, and then obtain critical conditions for any strategy to be evolutionarily stable on temporal networks. Interestingly, the re-scaled payoff matrix is a linear combination of the original payoff matrix with an additional one describing local competitions between any pair of different strategies, whose weights are solely determined by network topology and selection intensity. As a particular example, we apply the deterministic theory to analysing the impacts of temporal networks in the mini-ultimatum game, and find that temporally networked population structures result in the emergence of fairness. Our work offers theoretical insights into the subtle effects of network temporality on evolutionary game dynamics.

## 1. Introduction

Traditional evolutionary game theory studies the deterministic macro-behaviours of evolving populations under the infinitely well-mixed assumption in which individuals participate in strategic interactions [1,2]. In recent years, evolutionary game theory has been extended in numerous ways to explore dynamics in finite, structured, as well as unfix-sized populations owing to some realistic considerations [3–5]. For instance, stochastic theory has been developed for describing evolutionary game dynamics as a random process by considering the internal noise arising from the finiteness of the population [6]. It has been shown that the fluctuation effects owing to the transition of population size from infiniteness to finiteness may lead to fundamental changes of concepts in evolutionary game theory even in a well-mixed population, such as the conditions for evolutionary stability in the thermodynamic limit versus that in finite populations [7]. Meanwhile, evolutionary games in structured populations, as an interdisciplinary research field, have also attracted long-standing interests from applied mathematicians, statistical physicists, evolutionary biologists, theoretical economists and so on [8–12]. For evolutionary games on graphs, individuals occupy the nodes of a network, and the links of the network denote pairwise or collective relationships of interaction and replacement between individuals. In a seminal study, it has been found that spatial networks can result in evolutionary kaleidoscopes, deterministic chaos, as well as the stable co-existence between cooperators and defectors in the Prisoner’s Dilemma game [13]. Later studies from then on can be roughly classified into two categories: the general scenarios of natural selection that are related to many different research areas such as theoretical physics and the limit of weak selection which bridges the gap between evolutionary dynamics and population genetics. In the first class of studies, owing to the similar models and concepts with those in many-particle systems where the interacting individuals are also linked in a form of social networks, methods of statistical physics are widely employed to study pattern formation, phase transitions, equilibrium selection, and self-organization in evolutionary games on graphs [14–17]. In the second class of works that are more relevant to our study, evolutionary graph theory has been invented to investigate dynamics in graph-structured populations when evolution is affected by games merely with a linear perturbation [18–21]. Notably, an elegant rule has been found for the stochastic evolution of cooperation on regular graphs: if the benefit-to-cost ratio of an altruistic act exceeds the degree of the network, i.e., b/bcc>k , then natural selection on graphs favours the fixation of a cooperator [22]. Later on, this simple rule has been extended to any graph-based population structure: b/bcc>t2/t2(t3−t1)(t3−t1) where $t_{i},i\in \left\{1,2,3\right\}$ denotes the i -step coalescence time [23]. In the thermodynamic limit, it has been found that evolutionary games on regular graphs lead to a transformation of the original payoff matrix which quantitatively characterizes the impacts of network clustering among strategies on static graphs [24,25]. Moreover, we notice that evolutionary game dynamics are investigated not only on static graphs but also on adaptive networks that are coupled to the game dynamics by, e.g., adjusting links in accordance with the strategies of individuals [26,27].

Although great progress has been made on evolutionary games in structured populations, few studies until now have considered the population structures characterized by exogenous time-varying networks which are independent of the game dynamics upon them. Note that the underlying architectures of most realistic multi-agent systems are more suitably described as temporal networks where edges exist intermittently in comparison with the static counterpart in which edges are time-invariant. For instance, the biological links in metabolic networks refer to episodic chemical reactions between molecular species [28]; the friendship links in social networks are usually inferred from data of offline or online short-duration communications among human beings [29]. Until recently, a large body of analytical studies have already revealed that such temporality of edge activations in networks has significant impacts on many dynamical processes such as the spread of epidemic diseases and the diffusion of public opinions [30,31]. In this paper, we aim to develop a deterministic theory of evolutionary games on activity-driven time-varying networks since this network model has been widely studied as a paradigm for temporal networks [32,33]. Interestingly, we have derived replicator-like equations with a re-scaled payoff matrix which is a linear combination of the original payoff matrix with an additional one that quantitatively characterizes the impacts of competition between any pairs of distinct strategies. In addition, we have also obtained critical conditions for a strategy to be evolutionarily stable. Both parts of the deterministic theory, i.e. the replicator-like equations and the critical conditions for evolutionarily stable strategies, precisely describe the evolutionary dynamics of any pairwise games on temporal networks.

This paper is organized as follows. Section 2 describes our mathematical model. In §3, we develop a deterministic evolutionary game theory on temporal networks in the limit of weak selection. Section 4 is devoted to the application of the developed theory to explore the impacts of temporal networks in the evolutionary game dynamics of fairness. Discussion and conclusions are drawn in §5.

## 2. The mathematical model

Consider a symmetric game among $n\in N_{+}\setminus 1$ strategies, $s_{1},s_{2},\cdots ,s_{n}$ , with the general payoff matrix:


(2.1) $$A=\begin{array}{ccccc} & s_{1} & s_{2} & \cdots & s_{n}\\ s_{1} & a_{s_{1},s_{1}} & a_{s_{1},s_{2}} & \cdots & a_{s_{1},s_{n}}\\ s_{2} & a_{s_{2},s_{1}} & a_{s_{2},s_{2}} & \cdots & a_{s_{2},s_{n}}\\ \vdots & \vdots & \vdots & \ddots & \vdots \\ s_{n} & a_{s_{n},s_{1}} & a_{s_{n},s_{2}} & \cdots & a_{s_{n},s_{n}} \end{array},$$

where $a_{s_{i},s_{j}}\in R$ ( i,j∈{1,2,⋯,n} ) denotes the payoff for the row player with strategy $s_{i}$ versus another column player with strategy $s_{j}$ .

**Figure 1.**
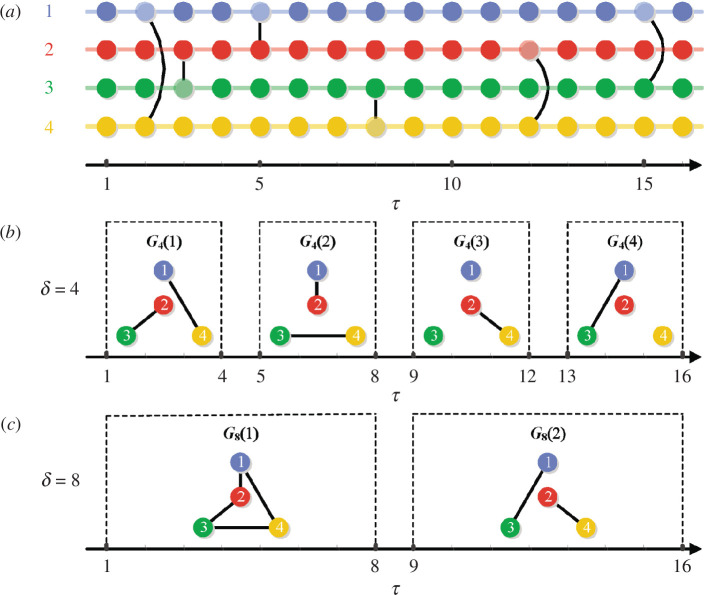
Example of an activity-driven temporal network for $a_{u}=0.1,u\in \left\{1,2,3,4\right\}$ , and m=1 . Panel (*a*) shows an instantaneous sequence of pairwise interactions between four players indicated by solid circles with different colours (player 1 : blue; player 2 : red; player 3 : green; player 4 : yellow) in the temporal dimension ranging from τ=1 to τ=16 . In this example, the solid circles with saturated (unsaturated) colours denote inactive (active) individuals, whereas each longitudinal black solid line connecting two nodes represents one instantaneous interaction at some time step τ . Panels (*b*) and (*c*) visualize dynamical processes of the integrated networks $G_{\delta }\left(t\right)$ for the time span δ=4 and δ=8 , respectively.

Individuals interact with each other in an infinite population structured by a temporal network G(τ)=(V,E(τ)) . Here, V denotes the set of nodes (or individuals) which is kept constant during evolution, while E(τ) the set of time-varying edges which describe the ever-changing activity-driven patterns of pairwise interactions between individuals. In the activity-driven model of temporal networks, each individual, e.g., u , is assigned by an activity potential $a_{u}\in \left(0,1\right]$ drawn from a probability density f(a) [32]. The activity rate $a_{u}$ characterizes the probability for the individual u to generate new interactions with other individuals per unit time. If it is activated, the individual u creates m undirected links each of which connects to a random selected individual regardless of its state, active or inactive (see figure 1*a*). Otherwise, the individual u is inactive but can also receive connections from other active individuals. Here, multiple edges or self-links are prohibited. After the transient interactions, all edges are discarded. The subsequent time steps of constructing and destructing the activity-driven instantaneous links follow exactly the same procedure as stated above. Then one can define the integrated network $G_{\delta }$ as the union of all instantaneous networks in the time duration τ∈[(t−1)δ+1,tδ] for $\tau ,t,\delta \in N_{+}$ :


(2.2) $$G_{\delta }\left(t\right)=\overset{\tau =t\delta }{\underset{\tau =\left(t-1\right)\delta +1}{⋃}}G\left(\tau \right),$$

where δ is the number of instantaneous networks generated for the time sequence τ∈{(t−1)δ+1,(t−1)δ+2,⋯,tδ} (see figure 1*b,c*). Noteworthy, if individuals in our model play the pairwise game with all their neighbours defined by the activity-driven temporary links of the δ successively generated instantaneous networks, then this is equivalent to the case that individuals interact with their immediate neighbours defined by the integrated network $G_{\delta }$ in the limit of an infinite population size, in which case the events of multiple edges or self-links rarely occur. In addition, t represents the time step for the evolutionary game process, each of which incorporates two consecutive procedures: game playing phase and strategy updating phase. In the stage of game playing, each individual accumulates its payoff by playing the pairwise game with its all neighbours, if any, on the integrated network $G_{\delta }\left(t\right)$ . Afterwards, all individuals enter into the stage of strategy updating by consideration of revising their strategies through imitating one of their neighbours, if any. Specifically, each individual will randomly select one of its neighbours on the integrated network $G_{\delta }\left(t\right)$ if co-players exist in its immediate neighbourhood. For example, the focal player, e.g. u , will imitate the strategy of a randomly chosen neighbour, e.g. v , with the probability given by the Fermi function:


(2.3) $$F\left(P_{v}-P_{u}\right)=\frac{1}{1+\exp\left(-\beta \left(P_{v}-P_{u}\right)\right)},$$

where β represents the intensity of natural selection, and $P_{u}$ and $P_{v}$ denote the accumulated payoffs of the focal individual u and the neighbouring role model v , respectively. Throughout this paper, we study the limiting case of weak selection β→0 , in which case equation (2.3) becomes


(2.4) $$F\left(P_{v}-P_{u}\right)=\frac{1}{2}+\frac{P_{v}-P_{u}}{4}\beta +O\left(\beta^{2}\right).$$

Note that study of the weak selection limit is relevant in a broad background of research fields including population genetics [34] and evolutionary dynamics [3], to name just a few.

## 3. Deterministic theory

Let us now develop a deterministic theory for the general case of n×n evolutionary games on temporal networks including derivation of the replicator-like equations and deduction of the critical conditions for evolutionary stability.

### 3.1. Replicator-like equations

Traditionally, replicator equations are employed to describe deterministic dynamics of evolutionary games in the infinite and well-mixed populations [35,36]. Here, the well-mixed assumption means that all individuals are equally likely to interact with each other. That is, an individual adopting strategy $s_{i}$ plays a game against another one adopting strategy $s_{j}$ with the probability given by $x_{s_{j}}\left(t\right)$ . Herein, $x_{s_{j}}\left(t\right)\in \left[0,1\right]$ , which satisfies $\sum_{j=1}^{n}x_{s_{j}}\left(t\right)=1$ , denotes the density of strategy $s_{j}$ at time step t . Thus, the average payoff of an individual with strategy $s_{i}$ at time step t is given by $\sum_{j=1}^{n}a_{s_{i}s_{j}}x_{s_{j}}\left(t\right)=e_{i}\cdot Ax\left(t\right)$ , where $e_{i}$ is the i th unit row vector, $x\left(t\right)={\left[x_{s_{1}}\left(t\right),x_{s_{2}}\left(t\right),\cdots ,x_{s_{n}}\left(t\right)\right]}^{T}$ , and the dot represents the inner product. The replicator equations can thus be expressed as


(3.1) $${\dot{x}}_{s_{i}}\left(t\right)=x_{s_{i}}\left(t\right)\left(e_{i}\cdot Ax\left(t\right)-x\left(t\right)\cdot Ax\left(t\right)\right),i\in \left\{1,2,\cdots ,n\right\},$$

where $\sum_{i,j=1}^{n}a_{s_{i}s_{j}}x_{s_{i}}\left(t\right)x_{s_{j}}\left(t\right)=\sum_{i=1}^{n}\left(e_{i}\cdot Ax\left(t\right)\right)x_{s_{i}}\left(t\right)=x\left(t\right)\cdot Ax\left(t\right)$ is the average payoff of the infinite and well-mixed population. If, however, the structure of the infinite population is organized by activity-driven temporal networks, we will show that the master equations governing the evolutionary game dynamics also have the form of replicator equations:


(3.2) $${\dot{x}}_{s_{i}}\left(t\right)=x_{s_{i}}\left(t\right)\left(e_{i}\cdot A^{\prime }x\left(t\right)-x\left(t\right)\cdot A^{\prime }x\left(t\right)\right),i\in \left\{1,2,\cdots ,n\right\},$$

where the transformed payoff matrix $A^{\prime }$ is a linear combination of the original payoff matrix A with an additional payoff matrix B which reflects the impacts of temporally networked population structure in the evolutionary game dynamics. Here, the additional payoff matrix B is calculated from the original payoff matrix A , and satisfies x(t)⋅Bx(t)=0 which indicates that the average payoff of the population remains the same as that in the standard replicator equations (for details, see below).

Let $x_{a,s_{i}}\left(t\right)$ denote the density of individuals with strategy $s_{i}$ and activation rate a at time step t . One can thus define the k th-order variable $I_{s_{i}}^{k}\left(t\right)$ related to a as


(3.3) $$I_{s_{i}}^{k}\left(t\right)=\int x_{a,s_{i}}\left(t\right)a^{k}da,k\in N.$$

Note that the zeroth-order variable $I_{s_{i}}^{0}\left(t\right)=\int x_{a,s_{i}}\left(t\right)da$ denotes the density of players adopting the strategy $s_{i}$ at time step t , i.e., $x_{s_{i}}\left(t\right)$ . This allows us to characterize the evolutionary game process on activity-driven temporal networks by the mean-field evolution of $I_{s_{i}}^{k}\left(t\right)$ :


(3.4) $$\begin{array}{l} {\dot{I}}_{s_{i}}^{k}\left(t\right)=-\sum_{j=1}^{n}\left(\int x_{a,s_{i}}\left(t\right)a^{k+1}da\right)\left(\int x_{a^{\prime },s_{j}}\left(t\right)da^{\prime }\right)F\left(E\left(P_{a^{\prime },s_{j}}\right)-E\left(P_{a,s_{i}}\right)\right)\\ \;-\sum_{j=1}^{n}\left(\int x_{a,s_{i}}\left(t\right)a^{k}da\right)\left(\int x_{a^{\prime },s_{j}}\left(t\right)a^{\prime }da^{\prime }\right)F\left(E\left(P_{a^{\prime },s_{j}}\right)-E\left(P_{a,s_{i}}\right)\right)\\ \;+\sum_{j=1}^{n}\left(\int x_{a,s_{j}}\left(t\right)a^{k+1}da\right)\left(\int x_{a^{\prime },s_{i}}\left(t\right)da^{\prime }\right)F\left(E\left(P_{a^{\prime },s_{i}}\right)-E\left(P_{a,s_{j}}\right)\right)\\ \;+\sum_{j=1}^{n}\left(\int x_{a,s_{j}}\left(t\right)a^{k}da\right)\left(\int x_{a^{\prime },s_{i}}\left(t\right)a^{\prime }da^{\prime }\right)F\left(E\left(P_{a^{\prime },s_{i}}\right)-E\left(P_{a,s_{j}}\right)\right), \end{array}$$

where the transition probabilities are given by


(3.5) $$\left\{ \begin{array}{l} F\left(E\left(P_{a^{\prime },s_{j}}\right)-E\left(P_{a,s_{i}}\right)\right)=\frac{1}{2}+\frac{E\left(P_{a^{\prime },s_{j}}\right)-E\left(P_{a,s_{i}}\right)}{4}\beta +O\left(\beta^{2}\right),\\ F\left(E\left(P_{a^{\prime },s_{i}}\right)-E\left(P_{a,s_{j}}\right)\right)=\frac{1}{2}+\frac{E\left(P_{a^{\prime },s_{i}}\right)-E\left(P_{a,s_{j}}\right)}{4}\beta +O\left(\beta^{2}\right). \end{array}\right.$$

Here, the expected payoff $E\left(P_{a,s_{i}}\right)$ of a player with activity rate a and strategy $s_{i}$ can be expressed as


(3.6) $$E\left(P_{a,s_{i}}\right)=\left(\left(a+\left\langle a\right\rangle \right)m\delta -1\right)\left(\sum_{l=1}^{n}a_{s_{i}s_{l}}I_{s_{l}}^{0}\left(t\right)\right)+a_{s_{i}s_{j}},$$

where (a+⟨a⟩)mδ=limN→+∞⁡N(1−e−amδ/amδNN)+⟨a⟩mδe−amδ/amδNN denotes the average degree of players with activity rate a accumulated during the time span τ∈[(t−1)δ+1,tδ] in the thermodynamic limit. On the right-hand side of equation (3.4), the first and second terms in the summation notations represent the overall decrease of the k th-order variable $I_{s_{i}}^{k}\left(t\right)$ in the mean-field limit owing to the imitation event where an active player with strategy $s_{i}$ adopts the strategy of another inactive player with strategy $s_{j}$ as well as to that in which a player with strategy $s_{i}$ imitates another player with strategy $s_{j}$ through the link generated by the role model, respectively. The third and fourth terms in the summation notations on the right side of equation (3.4) are derived, respectively, by following the same ideas as that in the first and second terms, but the only difference lies in that the players with strategy $s_{i}$ act as the role models in this case. From equation (3.4), one can derive the following differential equations for the higher order variables $I_{s_{i}}^{k}\left(t\right)$ ( $k\in N_{+}$ ):


(3.7) $$\begin{array}{l} {\dot{I}}_{s_{i}}^{k}(t)=\frac{1}{2}\left(-I_{s_{i}}^{k+1}(t)-\langle a\rangle I_{s_{i}}^{k}(t)+\langle a^{k}\rangle I_{s_{i}}^{1}(t)+\langle a^{k+1}\rangle I_{s_{i}}^{0}(t)\right)+O(\beta )\\ \;=G(I_{s_{i}}^{0}(t),I_{s_{i}}^{1}(t),I_{s_{i}}^{k}(t),I_{s_{i}}^{k+1}(t))+O(\beta ), \end{array}$$

where $\left\langle a^{k}\right\rangle$ ( $k\in N_{+}$ ) denotes the k th-order moment of the random variable a . Equation (3.7) indicates that the higher-order variables $I_{s_{i}}^{k}\left(t\right)$ ( $k\in N_{+}$ ) equilibrate much faster than the zeroth-order variable $I_{s_{i}}^{0}\left(t\right)$ in the limit of weak selection. In other words, the dynamics of $I_{s_{i}}^{k}\left(t\right)$ ( $k\in N_{+}$ ) can be decoupled from that of $I_{s_{i}}^{0}\left(t\right)$ by assuming that the zeroth-order variable does not change its value until the higher-order variables have reached their respective equilibria owing to a separation of time scales. As the dynamical system rapidly converges onto the slow manifold, defined by $G\left(I_{s_{i}}^{0}\left(t\right),I_{s_{i}}^{1}\left(t\right),I_{s_{i}}^{k}\left(t\right),I_{s_{i}}^{k+1}\left(t\right)\right)=0$ , we thus have


(3.8) $$I_{s_{i}}^{k+1}(t)+\langle a\rangle I_{s_{i}}^{k}(t)=\langle a^{k}\rangle I_{s_{i}}^{1}(t)+\langle a^{k+1}\rangle I_{s_{i}}^{0}(t),k\in N.$$

Note that equation (3.8) is also naturally satisfied for k=0 . From equation (3.8), the equilibrium values of the k th-order variable $I_{s_{i}}^{k}{\left(t\right)}^{*}$ can thus be derived as


(3.9) $$I_{s_{i}}^{k}(t{)}^{*}=\langle a^{k}\rangle I_{s_{i}}^{0}(t{)}^{*},k\in N_{+}.$$

Then we obtain the master equation that describes the evolutionary dynamics of $x_{s_{i}}\left(t\right)$ by substituting equation (3.9) into equation (3.4):


(3.10) $${\dot{x}}_{s_{i}}\left(t\right)=x_{s_{i}}\left(t\right)\left(e_{i}\cdot \left(\alpha_{1}A+\alpha_{2}B\right)x\left(t\right)-x\left(t\right)\cdot \left(\alpha_{1}A+\alpha_{2}B\right)x\left(t\right)\right)+O\left(\beta^{2}\right),$$

where the unnormalized weighted factors for the payoff matrices A and B are, respectively, given by


(3.11) $$\{\begin{array}{l} \alpha_{1}=\beta \left(3m\delta {\left\langle a\right\rangle }^{2}+m\delta \left\langle a^{2}\right\rangle -2\left\langle a\right\rangle \right)/2,\\ \alpha_{2}=\beta \left\langle a\right\rangle . \end{array}$$

The elements of the additional payoff matrix $B={\left(b_{s_{i}s_{j}}\right)}_{n\times n}$ are


(3.12) $$b_{s_{i}s_{j}}=a_{s_{i}s_{j}}-a_{s_{j}s_{i}}.$$

By neglecting $\beta^{2}$ and higher-order terms, we obtain replicator-like equations on temporal networks:


(3.13) $${\dot{x}}_{s_{i}}\left(t\right)=x_{s_{i}}\left(t\right)\left(e_{i}\cdot \left(\alpha_{1}A+\alpha_{2}B\right)x\left(t\right)-x\left(t\right)\cdot \left(\alpha_{1}A+\alpha_{2}B\right)x\left(t\right)\right),i\in \left\{1,2,\cdots ,n\right\}.$$

**Figure 2.**
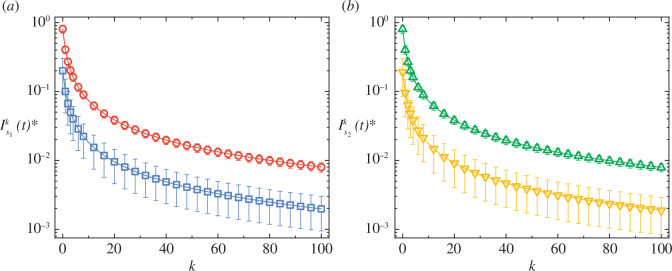
Deterministic dynamics of evolutionary games on temporal networks. Panels (*a*) and (*b*), respectively, present the equilibrium values of $I_{s_{1}}^{k}{\left(t\right)}^{*}$ for strategy $s_{1}$ (blue squares: $I_{s_{1}}^{0}\left(0\right)=0.2$ ; red circles: $I_{s_{1}}^{0}\left(0\right)=0.8$ ) and $I_{s_{2}}^{k}{\left(t\right)}^{*}$ for the other strategy $s_{2}$ (green triangles: $I_{s_{2}}^{0}\left(0\right)=1-I_{s_{1}}^{0}\left(0\right)=0.8$ ; yellow inverted triangles: $I_{s_{2}}^{0}\left(0\right)=1-I_{s_{1}}^{0}\left(0\right)=0.2$ ) as a function of the order k for a 2×2 pairwise game with the elements of payoff matrix A given by $a_{s_{1}s_{1}}=1$ , $a_{s_{1}s_{2}}=0$ , $a_{s_{2}s_{1}}=0$ , and $a_{s_{2}s_{2}}=0.5$ . The solid lines in both panels correspond to theoretical results predicted by equations (3.9) and (3.13), which perfectly coincide with the hollow symbols referring to simulation results obtained through the usage of agent-based modelling techniques. All data points are collected by averaging over 250 time steps after a relaxation process of 750 time steps with 200 different initial conditions of strategy distributions in total. Error bars indicate the standard deviation. Note that the longitudinal axes of both panels are logarithmic. Other parameter settings: f⁢(a)=1000⁢/⁢999 for a∈[0.001,1] and f(a)=0 otherwise, m=4 , δ=2 , $\beta =10^{-6}$ and population size $N=10^{4}$ .

Note that equation (3.13) accurately describes the deterministic dynamics of evolutionary games on activity-driven temporal networks in the limit of weak selection as equation (3.9) is derived without the usage of any conditions for moment closure. Indeed, we have shown in figure 2 that the combination of equation (3.9) with equation (3.13) precisely predicts the stationary values of the k th-order variables $I_{s_{i}}^{k}{\left(t\right)}^{*}$ , which were obtained through Monte Carlo simulations, even when the order k becomes considerably large. In addition, figure 2 also demonstrates that the equilibrium value of $I_{s_{i}}^{k}{\left(t\right)}^{*}$ is, as expected, super-linearly decreased with the order k , which indicates that the techniques of moment closure, though unnecessary in our work, can also be applicable to this study. On the other hand, we would like to emphasize that the deterministic descriptions of evolutionary game dynamics on temporal networks by equation (3.13) are valid only if the population size is sufficiently large. Owing to the reduction in population size, one can find that the level of internal noise arising from the finiteness of population size becomes even higher, which in turn leads to the extinction or fixation of some strategies because of the even larger fluctuations around population equilibria in the Monte Carlo simulations (see electronic supplementary material, figure S3*a,b*). In addition, we also observe that the uniform distribution of activity potential for m=4 and δ=2 leads to the formation of Gaussian-like degree distribution, of considerably weak clustering effects among nodes, as well as of relatively short average path length for the integrated network $G_{\delta }$ (see electronic supplementary material, figure S3*c–f*; for more detailed results on structural properties of temporal networks, see relevant references such as [30,32,37]).

### 3.2. Evolutionary stability

In evolutionary game theory, a fundamental concept is evolutionary stability [38]. The evolutionarily stable strategy refers to such a strategy that its monomorphic population rejects the invasion of any other mutant strategy. Consider a pairwise game defined by equation (2.1), then a strategy $s_{i}$ in an infinite and well-mixed population is an evolutionarily stable strategy if and only if


(3.14) $$a_{s_{i}s_{i}}>a_{s_{j}s_{i}}\;or\;a_{s_{i}s_{i}}=a_{s_{j}s_{i}}\;and\;a_{s_{i}s_{j}}>a_{s_{j}s_{j}}\;for\;any\;j\in N_{+}\setminus i.$$

Next, we characterize evolutionary stability on temporal networks by asking whether rare mutant strategies have an evolutionary advantage over the resident strategy. It has already been shown above that the impacts of edge temporality in the deterministic dynamics of evolutionary games can be quantitatively captured by a transformation of the original payoff matrix A . Particularly, the transformed payoff matrix $A^{\prime }$ is of the form:


(3.15) $$A^{\prime }=\begin{array}{ccccc} & s_{1} & s_{2} & \cdots & s_{n}\\ s_{1} & \alpha_{1}a_{s_{1},s_{1}} & (\alpha_{1}+\alpha_{2})a_{s_{1},s_{2}}-\alpha_{2}a_{s_{2},s_{1}} & \cdots & (\alpha_{1}+\alpha_{2})a_{s_{1},s_{n}}-\alpha_{2}a_{s_{n},s_{1}}\\ s_{2} & (\alpha_{1}+\alpha_{2})a_{s_{2},s_{1}}-\alpha_{2}a_{s_{1},s_{2}} & \alpha_{1}a_{s_{2},s_{2}} & \cdots & (\alpha_{1}+\alpha_{2})a_{s_{2},s_{n}}-\alpha_{2}a_{s_{n},s_{2}}\\ \vdots & \vdots & \vdots & \ddots & \vdots \\ s_{n} & (\alpha_{1}+\alpha_{2})a_{s_{n},s_{1}}-\alpha_{2}a_{s_{1},s_{n}} & (\alpha_{1}+\alpha_{2})a_{s_{n},s_{2}}-\alpha_{2}a_{s_{2},s_{n}} & \cdots & \alpha_{1}a_{s_{n},s_{n}} \end{array}.$$

Once such a transformation is performed, then evolutionary game dynamics on temporal networks is governed by a set of replicator-like equations as it does in the infinitely well-mixed populations (compare equation (3.1) with equation (3.13)). Therefore, one can obtain the following conditions for a strategy $s_{i}$ to be an evolutionarily stable strategy according to the transformed payoff matrix $A^{\prime }$ (see equation (3.15)):


(3.16) $$\alpha_{1}a_{s_{i}s_{i}}+\alpha_{2}a_{s_{i}s_{j}}>\left(\alpha_{1}+\alpha_{2}\right)a_{s_{j}s_{i}}\;for\;any\;j\in N_{+}\setminus i.$$

Note that the conditions given by equation (3.16) is sufficient because we do not discuss the evolutionary stability of $s_{i}$ for the ungeneric case $\alpha_{1}a_{s_{i}s_{i}}+\alpha_{2}a_{s_{i}s_{j}}=\left(\alpha_{1}+\alpha_{2}\right)a_{s_{j}s_{i}}$ .

## 4. Numerical example

In order to show the impacts of temporally networked population structures in evolutionary game dynamics, let us now apply the deterministic theory to studying the emergence of fairness in the mini-ultimatum game. In the ultimatum game, two players are given a chance to win a certain sum of money. The strategy of each player is characterized by a vector [p,q] , where p∈[0,1] denotes the fraction of the money proposed by the player when acting as a proposer, and q∈[0,1] represents the acceptance threshold, i.e., the minimum fraction of the money that the player agrees to accept when acting as a responder [39]. Instead of studying the full ultimatum game with its continuum of strategies, we here consider the so-called mini-ultimatum game in which only two possible offer and acceptance levels h (i.e., high level) and l (i.e., low level) satisfying 0<l<h<1/122 are present [40]. Accordingly, there are four different strategies in such a mini-ultimatum game: (i) reasonable strategy [l,l] which offers little to the responder and also accepts low offers as a responder (*R*); (ii) generous strategy [h,l] which makes high offers but is willing to accept a low offer (*G*); (iii) fair strategy [h,h] which offers and demands high shares (*F*); (iv) immoral strategy [l,h] which offers little but only accepts a high share (*I*). The payoff matrix of the mini-ultimatum game thus is


(4.1) $$A_{MUG}=\begin{array}{ccccc} & R & G & F & I\\ R & 1 & 1-l+h & h & l\\ G & 1-h+l & 1 & 1 & 1-h+l\\ F & 1-h & 1 & 1 & 1-h\\ I & 1-l & 1-l+h & h & 0 \end{array}.$$

In the mini-ultimatum game, the standard replicator equations predict that the absorbing state ${\left[x_{R},x_{G},x_{F},x_{I}\right]}^{T}={\left[0,0,0,1\right]}^{T}$ is unstable and that all evolutionary trajectories in the interior of the simplex $S_{4}$ will converge to the boundary face of $S_{4}$ on which the frequency of immoral strategy $x_{I}=0$ . On this face, there exists no equilibrium mixed state among reasonable strategy R , generous strategy G , and fair strategy F . All points on the edge G−F of $S_{4}$ are fixed points, those of which between F and [0,(1−h)/(1−h)(1−l)(1−l),(h−l)/(h−l)(1−l)(1−l),0]T are stable and the rest of which are unstable and can be invaded by R and I . On the edge R−G , the reasonable strategy dominates the general strategy. The edge R−F is bi-stable between the reasonable strategy and the fair strategy with one unstable interior fixed point located at ${\left[1-h,0,h,0\right]}^{T}$ . In addition, there also exists an interior fixed point ${\left[0,1-h+l,0,h-l\right]}^{T}$ which is stable on the edge G−I but can be invaded by the reasonable or fair strategy. Therefore, the evolutionary orbits of the simplex $S_{4}$ can converge either to R or to the segment between F and [0,(1−h)/(1−h)(1−l)(1−l),(h−l)/(h−l)(1−l)(1−l),0]T . However, random shocks may lead to neutral drift of the population locating at the stable segment towards the unstable segment between [0,(1−h)/(1−h)(1−l)(1−l),(h−l)/(h−l)(1−l)(1−l),0]T and G , which eventually results in the complete dominance of the reasonable strategy R . In sum, traditional evolutionary game theory forecasts the rational solution that the proposer offers the low share and the responder accepts it in the mini-ultimatum game, which means that the stabilization of unfairness is resulted in the infinitely well-mixed populations [40].

**Figure 3.**
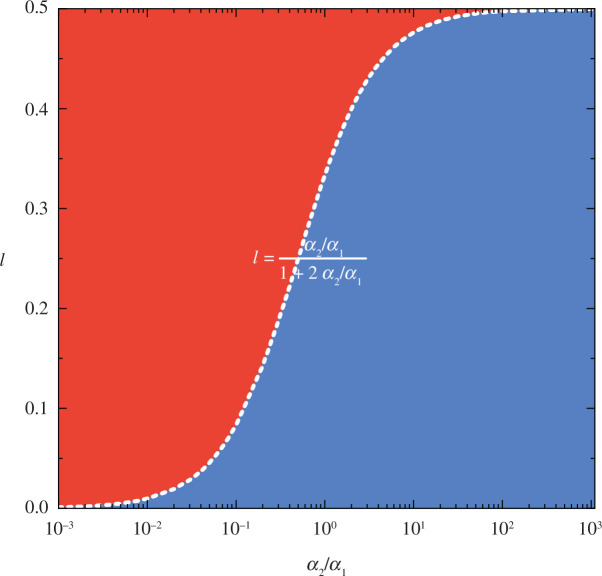
Phase separation for evolutionary stability of the reasonable strategy R on the two-dimensional α2/α2α1α1−l parameter space. The phase boundary given by l=α2/α2(α1+2α2)(α1+2α2) divides the two-dimensional α2/α2α1α1−l parameter plane into two parts: (i) the upper part wherein the reasonable strategy R is evolutionarily stable (red area); (ii) the bottom part wherein the reasonable strategy R is evolutionarily unstable (blue area). Note that the horizontal axis is logarithmic.

The evolutionary game dynamics is dramatically changed if one considers the mini-ultimatum game in a temporally network-structured population. In this case, we find from equations (3.16) and (4.1) that the reasonable strategy R becomes evolutionarily unstable if the following critical condition is satisfied (see figure 3):


(4.2) $$l<\frac{\alpha_{2}}{\alpha_{1}+2\alpha_{2}}.$$

**Figure 4.**
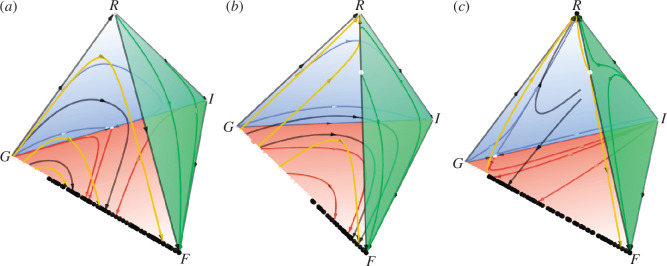
Phase portraits of the evolutionary mini-ultimatum game on temporal networks in the simplex $S_{4}$ for different combinations of h and l when $\alpha_{1}=1$ and $\alpha_{2}=2$ : (*a*) h=0.3 and l=0.2 , where h and l satisfy l<h<α2/α2(α1+2α2)(α1+2α2) and h−l<α1/α1(α1+2α2)(α1+2α2) ; (*b*) h=0.45 and l=0.2 , in which h and l satisfy l<α2/α2(α1+2α2)(α1+2α2) and h−l>α1/α1(α1+2α2)(α1+2α2) ; (*c*) h=0.45 and l=0.42 , wherein h and l satisfy h>l>α2/\gtl>α2(α1+2α2) and h−l<α1/α1(α1+2α2)(α1+2α2) . The arrows show the directions of natural selection, and the black (white) circles denote asymptotically stable (unstable) fixed points. Each face of the simplex $S_{4}$ is drawn with different colours: the R−G−F face (completely transparent yellow); the R−G−I face (partially transparent blue); the R−F−I face (partially transparent green); the G−F−I face (partially transparent red). For the purpose of clarity, evolutionary orbits started from the interior points of each face are marked with the same colour as their respective background colour, whereas those origined from the interior points of the simplex $S_{4}$ or of its edges are coloured with black. Note that all evolutionary trajectories will eventually converge to the fair strategy F in panels (*a*) and (*b*) but will move towards the reasonable strategy R in panel (*c*) if one further considers the presence of small random mutations in the population. All graphical outputs are produced by the Dynamo software [42].

Note that equation (4.2) also guarantees that the fair strategy F is the unique pure stable state of the evolutionary mini-ultimatum game on temporal networks. Interestingly, we have found that the transformed payoff matrix ${A^{\prime }}_{MUG}$ inherits the property of the original payoff matrix $A_{MUG}$ that $a_{s,R}^{\prime }+a_{s,F}^{\prime }=a_{s,G}^{\prime }+a_{s,I}^{\prime }$ for s∈{R,G,F,I} , and therefore, $E\,\left(P_{R}\right)+E\,\left(P_{F}\right)=E\,\left(P_{G}\right)+E\,\left(P_{I}\right)$ holds for any composition of the population. This indicates that the replicator-like dynamics admits a constant of motion given by (xRxF)/(xRxF)(xGxI)(xGxI)=K with K∈(0,+∞) [41]. As a result, the interior space of the simplex $S_{4}$ collapses into the invariant manifolds $W_{K}$ corresponding to saddle-like surfaces spanned by the frame R−G−F−I−R , where R , G , F , and I represent the vertexes of $S_{4}$ satisfying $x_{R}=1$ , $x_{G}=1$ , $x_{F}=1$ , and $x_{I}=1$ , respectively. Moreover, in the interior of $S_{4}$ , we have $E\,\left(P_{R}\right)-E\,\left(P_{I}\right)=E\,\left(P_{G}\right)-E\,\left(P_{F}\right)=\left[\left(\alpha_{1}+2\,\alpha_{2}\right)\,l-\alpha_{2}\right]\,\left(x_{R}+x_{I}\right)$ , which means that xR/xRxIxI and xG/xGxFxF are synchronously increasing if l>α2/α2(α1+2α2)(α1+2α2) but are synchronously decreasing if l<α2/α2(α1+2α2)(α1+2α2) . Therefore, there is no fixed point in the interior of $S_{4}$ . Along the boundary of the simplex $S_{4}$ , each point on the edge G−F is a rest point, which is stable if it locates between F and [0,(α1(1−h)+α2(1−2h))/(α1(1−h)+α2(1−2h))(α1(1−l)+α2(1−2l))(α1(1−l)+α2(1−2l)),(α1(h−l)+2α2(h−l))/(α1(h−l)+2α2(h−l))(α1(1−l)+α2(1−2l))(α1(1−l)+α2(1−2l)),0]T but is unstable otherwise (see figure 4). An unstable fixed point ${\left[0,\left(\alpha_{1}\,\left(1-h+l\right)-2\,\alpha_{2}\,\left(h-l\right)\right)\,/\,\alpha_{1},0,\left(\alpha_{1}\,\left(h-l\right)+2\,\alpha_{2}\,\left(h-l\right)\right)\,/\,\alpha_{1}\right]}^{T}$ emerges on the edge G−I only if h−l<α1/α1(α1+2α2)(α1+2α2) (compare figure 4*a* or 4*c* with figure 4*b*). On the edge R−I , the game dynamics alone the edge R−I is dependent on l (compare figure 4*a* or 4*b* with figure 4*c*): (i) if l<α2/α2(α1+2α2)(α1+2α2) , the evolutionary trajectory flows from R to I ; (ii) if l>α2/α2(α1+2α2)(α1+2α2) , the evolutionary direction reverses. If h∈(α2/α2(α1+2α2)(α1+2α2),1/122) , the reasonable strategy R and the fair strategy F are bi-stable on the edge R−F with the unique unstable fixed point [(α1(1−h)+α2(1−2h))/(α1(1−h)+α2(1−2h))α1α1,0,(α1h−α2(1−2h))/(α1h−α2(1−2h))α1α1,0]T determining their respective attraction basins (see figure 4*b,c*). Otherwise, the reasonable strategy R is completely dominated by the fair strategy F (see figure 4*a*). In short, temporal networks can promote the emergence of fairness by simultaneously stabilizing the fair strategy F as well as destabilizing the reasonable strategy R in the evolutionary mini-ultimatum game.

## 5. Discussion and conclusions

In summary, we have developed a deterministic theory for describing evolutionary dynamics of any n×n pairwise games in an infinite population whose structure is organized by activity-driven temporal networks. The replicator-like equations derived in the limit of weak selection reveal that the temporally networked population structure introduces a transformation of the original payoff matrix from A to $A^{\prime }=\alpha_{1}A+\alpha_{2}B$ (see equation (3.13)), where the additional payoff matrix B characterizes the local competition between any pair of different strategies and the unnormalized weights $\alpha_{1}$ and $\alpha_{2}$ quantify the importance of global and local interactions, respectively. Note further that B is an antisymmetric payoff matrix, i.e., $b_{s_{i}s_{j}}=-b_{s_{j}s_{i}}$ , which means that the gain of a strategy in a local competition is at the loss of another one—a type of strategic interaction called the zero-sum game (see equation (3.12)). Therefore, different from the previous study on the impacts of regular graphs in the deterministic dynamics of evolutionary games [24], temporal networks do not introduce any effects of network clustering among strategies, but rather the effects of spite between any pairs of different strategies. This is essential to understand the effects of temporally networked population structures on the emergence of fairness in the mini-ultimatum game (see §4). Moreover, one can find from equations (3.11) and (3.13) that the normalized weight α¯2=α2/α2(α1+α2)(α1+α2)=2⟨a⟩/2⟨a⟩(mδ(3⟨a⟩2+⟨a2⟩))(mδ(3⟨a⟩2+⟨a2⟩)) qualitatively determines the evolutionary game dynamics on temporal networks. Therefore, one can conclude that the deterministic dynamics of evolutionary games on temporal networks becomes more similar to that in well-mixed populations if the probability distribution of the activity potentials is more heterogeneous. Otherwise, local interactions described by the additional payoff matrix B have a stronger influence in the evolutionary game dynamics on temporal networks. On the other hand, we can also draw an expectable conclusion that the increment of either m or δ leads to an increasing reduction in the effects exerted by temporally networked population structure on the evolutionary game dynamics.

It should be noted that a few of previous studies have investigated the effects of temporal networks on evolutionary dynamics of pairwise social dilemma games with cooperation and defection as the only two competing strategies [43–46]. In the framework of deterministic evolutionary game dynamics, previous studies are carried out mainly through agent-based simulations on temporal networks which are constructed from empirical data sets [43,44]. Therefore, the developed method here can be used to verify and explain the fundamental role played by temporal networks on the evolution of cooperation from a theoretical perspective. For example, our results are consistent with [43,44] in that the cooperation level at the equilibrium is decreased with the ratio of timescale between strategy updating and network evolution (see electronic supplementary material, figure S2). Somewhat surprisingly, our theoretical predictions can even qualitatively reproduce their simulation results, as evidenced by a comparison between figure S1 in the electronic supplementary material and figs. 2 and 3 in [43], even though they are obtained under different assumptions on population size and selection strength. On the other hand, a general theory is still lacking for describing the evolutionary dynamics of multi-strategy games on temporal networks owing to the time-varying feature of network topology as well as the high-order correlations of game dynamics on graphs. Here, we provide the first theoretical description of evolutionary game processes for any n×n pairwise games on activity-driven temporal networks (see equation (3.4)), and also derive its analytical expressions in a closed form under the limiting condition of weak selection (see equation (3.13)). Finally, recent studies have also revealed that temporal networks in reality are characterized by burstiness, memory, as well as strong topological correlations [37,47]. In the future, it is necessary to extend the present framework to further consider all these important features. Works along this line are in progress.

## Data Availability

Source code for agent-based simulations in figure 2 is available from the OSF repository [48]. Electronic supplementary material is available online [49].

## References

[1] Maynard Smith J . 1982 Evolution and the theory of games. Cambridge, UK: Cambridge University Press. (10.1017/CBO9780511806292)

[2] Sandholm WH . 2010 Population games and evolutionary dynamics. Cambridge, MA: MIT Press.

[3] Nowak MA . 2006 Evolutionary dynamics: exploring the equation of life. Cambridge, MA: Harvard University Press. (10.2307/j.ctvjghw98)

[4] Huang W , Hauert C , Traulsen A . 2015 Stochastic game dynamics under demographic fluctuations. Proc. Natl Acad. Sci. USA **112** , 9064–9069. (10.1073/pnas.1418745112) 26150518 PMC4517200

[5] Wang G , Su Q , Wang L , Plotkin JB . 2023 Reproductive variance can drive behavioral dynamics. Proc. Natl Acad. Sci. USA **120** , e2216218120. (10.1073/pnas.2216218120) 36927152 PMC10041125

[6] Traulsen A , Hauert C . 2009 Stochastic evolutionary game dynamics. In Reviews of nonlinear dynamics and complexity (ed. HG Schuster ), pp. 25–62. Berlin, Germany: Wiley-VCH. (10.1002/9783527628001)

[7] Nowak MA , Sasaki A , Taylor C , Fudenberg D . 2004 Emergence of cooperation and evolutionary stability in finite populations. Nature **428** , 646–650. (10.1038/nature02414) 15071593

[8] Szabó G , Fáth G . 2007 Evolutionary games on graphs. Phys. Rep. **446** , 97–216. (10.1016/j.physrep.2007.04.004)

[9] Gross T , Blasius B . 2008 Adaptive coevolutionary networks: a review. J. R. Soc. Interface **5** , 259–271. (10.1098/rsif.2007.1229) 17971320 PMC2405905

[10] Nowak MA , Tarnita CE , Antal T . 2010 Evolutionary dynamics in structured populations. Philos. Trans. R. Soc. B **365** , 19–30. (10.1098/rstb.2009.0215) PMC2842709 20008382

[11] Perc M , Gómez-Gardeñes J , Szolnoki A , Floría LM , Moreno Y . 2013 Evolutionary dynamics of group interactions on structured populations: a review. J. R. Soc. Interface **10** , 20120997. (10.1098/rsif.2012.0997) 23303223 PMC3565747

[12] Battiston F , Cencetti G , Iacopini I , Latora V , Lucas M , Patania A , Young JG , Petri G . 2020 Networks beyond pairwise interactions: structure and dynamics. Phys. Rep. **874** , 1–92. (10.1016/j.physrep.2020.05.004)

[13] Nowak MA , May RM . 1992 Evolutionary games and spatial chaos. Nature **359** , 826–829. (10.1038/359826a0)

[14] Hauert C , Doebeli M . 2004 Spatial structure often inhibits the evolution of cooperation in the snowdrift game. Nature **428** , 643–646. (10.1038/nature02360) 15074318

[15] Chen X , Szolnoki A , Perc M . 2015 Competition and cooperation among different punishing strategies in the spatial public goods game. Phys. Rev. E **92** , 012819. (10.1103/PhysRevE.92.012819) 26274237

[16] Szolnoki A , Perc M . 2017 Second-order free-riding on antisocial punishment restores the effectiveness of prosocial punishment. Phys. Rev. X **7** , 041027. (10.1103/PhysRevX.7.041027)

[17] Perc M , Jordan JJ , Rand DG , Wang Z , Boccaletti S , Szolnoki A . 2017 Statistical physics of human cooperation. Phys. Rep. **687** , 1–51. (10.1016/j.physrep.2017.05.004)

[18] Lieberman E , Hauert C , Nowak MA . 2005 Evolutionary dynamics on graphs. Nature **433** , 312–316. (10.1038/nature03204) 15662424

[19] Fu F , Wang L , Nowak MA , Hauert C . 2009 Evolutionary dynamics on graphs: efficient method for weak selection. Phys. Rev. E **79** , 046707. (10.1103/PhysRevE.79.046707) PMC2735202 19518380

[20] Zhou L , Wu B , Du J , Wang L . 2021 Aspiration dynamics generate robust predictions in heterogeneous populations. Nat. Commun. **12** , 3250. (10.1038/s41467-021-23548-4) 34059670 PMC8166829

[21] Su Q , McAvoy A , Mori Y , Plotkin JB . 2022 Evolution of prosocial behaviours in multilayer populations. Nat. Hum. Behav. **6** , 338–348. (10.1038/s41562-021-01241-2) 34980900

[22] Ohtsuki H , Hauert C , Lieberman E , Nowak MA . 2006 A simple rule for the evolution of cooperation on graphs and social networks. Nature **441** , 502–505. (10.1038/nature04605) 16724065 PMC2430087

[23] Allen B , Lippner G , Chen YT , Fotouhi B , Momeni N , Yau ST , Nowak MA . 2017 Evolutionary dynamics on any population structure. Nature **544** , 227–230. (10.1038/nature21723) 28355181

[24] Ohtsuki H , Nowak MA . 2006 The replicator equation on graphs. J. Theor. Biol. **243** , 86–97. (10.1016/j.jtbi.2006.06.004) 16860343 PMC2430083

[25] Ohtsuki H , Nowak MA , Pacheco JM . 2007 Breaking the symmetry between interaction and replacement in evolutionary dynamics on graphs. Phys. Rev. Lett. **98** , 108106. (10.1103/PhysRevLett.98.108106) 17358573 PMC2387227

[26] Pacheco JM , Traulsen A , Nowak MA . 2006 Active linking in evolutionary games. J. Theor. Biol. **243** , 437–443. (10.1016/j.jtbi.2006.06.027) 16901509 PMC3279753

[27] Wu B , Zhou D , Wang L . 2011 Evolutionary dynamics on stochastic evolving networks for multiple-strategy games. Phys. Rev. E **84** , 046111. (10.1103/PhysRevE.84.046111) 22181231

[28] Almaas E , Kovács B , Vicsek T , Oltvai ZN , Barabási AL . 2004 Global organization of metabolic fluxes in the bacterium Escherichia coli. Nature **427** , 839–843. (10.1038/nature02289) 14985762

[29] Cohen R , Havlin S . 2010 Complex networks: structure, robustness and function. Cambridge, UK: Cambridge University Press. (10.1017/CBO9780511780356)

[30] Holme P , Saramäki J (eds). 2013 Temporal networks. Berlin, Germany: Springer. (10.1007/978-3-642-36461-7)

[31] Masuda N , Holme P (eds). 2017 Temporal network epidemiology. Singapore: Springer. (10.1007/978-981-10-5287-3)

[32] Perra N , Gonçalves B , Pastor-Satorras R , Vespignani A . 2012 Activity driven modeling of time varying networks. Sci. Rep. **2** , 469. (10.1038/srep00469) 22741058 PMC3384079

[33] Holme P , Saramäki J . 2012 Temporal networks. Phys. Rep. **519** , 97–125. (10.1016/j.physrep.2012.03.001)

[34] Kimura M . 1983 The neutral theory of molecular evolution. Cambridge, UK: Cambridge University Press. (10.1017/CBO9780511623486)

[35] Taylor PD , Jonker LB . 1978 Evolutionary stable strategies and game dynamics. Math. Biosci. **40** , 145–156. (10.1016/0025-5564(78)90077-9)

[36] Hofbauer J , Sigmund K . 1998 Evolutionary games and population dynamics. Cambridge, UK: Cambridge University Press. (10.1017/CBO9781139173179)

[37] Masuda N , Lambiotte R . 2020 A guide to temporal networks. Singapore: World Scientific. (10.1142/q0268)

[38] Weibull JW. 1995 Evolutionary game theory. Cambridge, MA: MIT Press.

[39] Güth W , Schmittberger R , Schwarze B . 1982 An experimental analysis of ultimatum bargaining. J. Econ. Behav. Org. **3** , 367–388. (10.1016/0167-2681(82)90011-7)

[40] Nowak MA , Page KM , Sigmund K . 2000 Fairness versus reason in the ultimatum game. Science **289** , 1773–1775. (10.1126/science.289.5485.1773) 10976075

[41] Sigmund K , Hauert C , Nowak MA . 2001 Reward and punishment. Proc. Natl Acad. Sci. USA **98** , 10757–10762. (10.1073/pnas.161155698) 11553811 PMC58548

[42] Franchetti F , Sandholm WH . 2013 An introduction to dynamo: diagrams for evolutionary game dynamics. Biol. Theory **8** , 167–178. (10.1007/s13752-013-0109-z)

[43] Cardillo A , Petri G , Nicosia V , Sinatra R , Gómez-Gardeñes J , Latora V . 2014 Evolutionary dynamics of time-resolved social interactions. Phys. Rev. E **90** , 052825. (10.1103/PhysRevE.90.052825) 25493851

[44] Li A , Zhou L , Su Q , Cornelius SP , Liu YY , Wang L , Levin SA . 2020 Evolution of cooperation on temporal networks. Nat. Commun. **11** , 2259. (10.1038/s41467-020-16088-w) 32385279 PMC7210286

[45] Sheng A , Li A , Wang L . 2023 Evolutionary dynamics on sequential temporal networks. PLoS Comput. Biol **19** , 1–19. (10.1371/journal.pcbi.1011333) PMC10434888 37549167

[46] Su Q , McAvoy A , Plotkin JB . 2023 Strategy evolution on dynamic networks. Nat. Comput. Sci. **3** , 763–776. (10.1038/s43588-023-00509-z) 38177777

[47] Sheng A , Su Q , Li A , Wang L , Plotkin JB . 2023 Constructing temporal networks with bursty activity patterns. Nat. Commun. **14** , 7311. (10.1038/s41467-023-42868-1) 37951967 PMC10640578

[48] Wang X , Fu F , Wang L . 2024 Source code: Deterministic theory of evolutionary games on temporal networks. OSF https://osf.io/3bj9m/ 10.1098/rsif.2024.0055 PMC11286197 38807526

[49] Wang X , Fu F , Wang L . 2024 Supplementary material from: Deterministic theory of evolutionary games on temporal networks. Figshare (10.6084/m9.figshare.c.7200887) PMC11286197 38807526

